# 5G as a wireless power grid

**DOI:** 10.1038/s41598-020-79500-x

**Published:** 2021-01-12

**Authors:** Aline Eid, Jimmy G. D. Hester, Manos M. Tentzeris

**Affiliations:** 1 School of Electrical and Computer Engineering, Georgia Institute of Technology, Atlanta, GA 30332 USA; 2 Atheraxon, Atlanta, GA 30308 USA

**Keywords:** Devices for energy harvesting, Electrical and electronic engineering

## Abstract

5G has been designed for blazing fast and low-latency communications. To do so, mm-wave frequencies were adopted and allowed unprecedently high radiated power densities by the FCC. Unknowingly, the architects of 5G have, thereby, created a wireless power grid capable of powering devices at ranges far exceeding the capabilities of any existing technologies. However, this potential could only be realized if a fundamental trade-off in wireless energy harvesting could be circumvented. Here, we propose a solution that breaks the usual paradigm, imprisoned in the trade-off between rectenna angular coverage and turn-on sensitivity. The concept relies on the implementation of a Rotman lens between the antennas and the rectifiers. The printed, flexible mm-wave lens allows robust and bending-resilient operation over more than 20 GHz of gain and angular bandwidths. Antenna sub-arrays, rectifiers and DC combiners are then added to the structure to demonstrate its combination of large angular coverage and turn-on sensitivity—in both planar and bent conditions—and a harvesting ability up to a distance of 2.83 m in its current configuration and exceeding 180 m using state-of-the-art rectifiers enabling the harvesting of several μW of DC power (around 6 μW at 180 m with 75 dBm EIRP).

## Introduction

Our era is witnessing a rapid development in the field of millimeter-wave (mm-wave) and Internet of Things (IoT) technologies with a projected 40 billion IoT devices to be installed by 2025^1^. This could result in a huge number of batteries needing to be continuously charged and replaced. The design and realization of energy-autonomous, self-powered systems: the perpetual IoT, is therefore highly desirable. One potential way of satisfying these goals is through electromagnetic energy harvesting. A powerful source for electromagnetic scavenging is mm-wave energy, present in the fifth-generation (5G) of mobile communications bands (above 24 GHz), where the limits of allowable transmitted Effective Isotropic Radiated Power (EIRP) by the Federal Communications Commission (FCC) regulations are pushed beyond (reaches 75 dBm) that of their lower-frequency counterparts. Following the path loss model defined by the 3rd Generation Partnership Project Technical Report 3GPP TR 38.901 (release 16) in outdoor Urban Macro Line of Sight conditions (UMa LOS), the power density expected to be received at 28 GHz for a transmitted power of 75 dBm EIRP is 28 μW cm^−2^ at a distance of 100 m away from the transmitter. This demonstrates the ability of 5G to create a usable network of wireless power. In addition to the advantage of high transmitted power available at 5G, moving to mm-wave bands allows the realization of modular antennas arrays instead of single elements, thereby allowing a fine scaling of their antenna aperture, which can more than compensate for the high path loss at these frequencies through the addition of extremely-large gains^2^. However, one limitation accompanies large gain antennas: their inability to provide a large angular coverage. As the relative orientations of the sources and harvesters are generally unknown, the use of large aperture mm-wave harvesters may seem limiting and impossible. Individual rectennas, constituted of small antenna elements, can realistically be DC combined. However, this approach does not increase the turn-on sensitivity (lowest turn-on power) of the overall rectenna system: RF combination is needed.

Beamforming networks (BFNs) are used to effectively create simultaneous beam angular coverage with large-gain arrays, by mapping a set of directions to a set of feeding ports. An important class of these multiple networks is the microwave passive BFN that has been widely used in switched-beam antenna systems and applications. Hybrid combination techniques, based on Butler matrix networks, have been used in previous works for energy harvesting at lower frequencies^3,4^,—more specifically at 2.45 GHz—to achieve wider angular coverage harvesting. However, these Ultra-High Frequency (UHF) arrays are impractically large for IoT applications and the implementation of their Butler matrices at higher frequencies would necessitate costly high-resolution fabrication. While sub-optimal—because of its large size—in the UHF band, the Rotman lens becomes the BFN of choice in the realm of mm-wave energy harvesting. Compared to their lower frequencies counterpart, fewer implementations are presented in the literature targeting energy harvesting at higher frequencies, more specifically 24 GHz and above. However, these systems later displayed in the table of comparison^5–7^, suffer from a narrow angular coverage.

In this paper, the authors demonstrate a full implementation of an entirely flexible, bending-resilient and simultaneously high gain and large angular coverage system for 5G/mm-wave energy harvesting based on a Rotman lens. For IoT applications, there is a benefit to making extremely low-profile devices that can conformally fit onto any surface in the environment such as walls, bodies, vehicles, etc. Therefore, thanks to the use of mm-waves, antennas with such features can be readily designed and fabricated. A Rotman lens-based rectenna has been first proposed in^8^, where a preliminary prototype and approach were presented, resulting in a quasi-flexible system, 80° angular coverage and 21-fold increase in the harvested power compared to a non-Rotman-based system. Here, the previously-predicted potential of 5G-powered nodes for the IoT and long-range passive mm-wave Radio Frequency IDentification (RFID) devices, is further taken advantage of, and effectively demonstrated. In order to do so, a thorough analysis of the lens itself—a structure that was not revealed in^8^—is first presented, exposing its key design parameters and resulting measured broadband behavior tested in both planar and bent conditions over more than 20 GHz of bandwidth. In addition, a scalability study of the approach, outlining the optimal size of such a system is reported, thereby proving the extent of the capability of providing a combination of good array factor and wide beam coverage. The novelty of this system also lies in the realization of a fully-flexible 28 GHz Rotman-lens-based rectenna system, completed by the design of a new DC combiner on a flexible 125 μm-thin polyimide Kapton substrate. The new DC combiner uses a reduced number of bypass diodes and increases the angular coverage of the system by more than 30% compared to^8^. Furthermore, the frequency-broadband behavior enabled by the use of the Rotman lens makes the full rectenna system bending-resilient, a property now demonstrated through its characterizations in flexing and conformally-mounted configurations. Finally, the system’s potential for long-range mm-wave harvesting is expressed for the first time, by reporting an unprecedented harvesting range of 2.83 m.

## Experiments, results and discussions

### Rotman lens scalability study for harvesting applications

**Figure 1:**
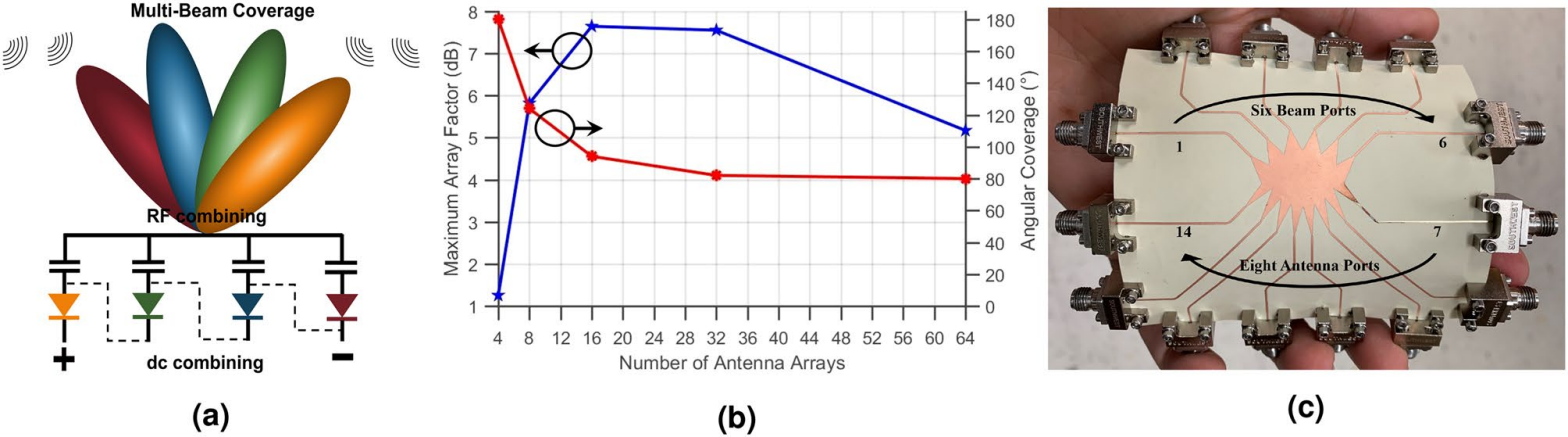
(**a**) Dual combining (RF + DC) enabled by the use of the Rotman lens between the antennas and the rectifiers, (**b**) plot of the simulated maximum array factors and angular coverages for different-size Rotman lenses and (**c**) picture of the fabricated Rotman lens structure.

The Rotman lens, introduced in the 1960s, constitutes one of the most common and cost-effective designs for BFNs and is commonly utilized to enable multibeam phased array system^9^ and wide-band operation, thanks to its implementation of true-time-delays^10^. By properly tuning the shape of the lens according to the geometrical optics approximation with the goal of focalizing plane waves impinging on the antenna side of the lens to different focal points on the beam-ports side of the lens, one achieves a lens-shaped structure with two angles of curvatures: one on the beam-ports side, and the other on the antenna side^11^. Because the lens is capable of focusing the energy coming from a given direction into its geometrically-associated beam port, the proposed scheme loads each of these ports with a rectifier, thereby channeling the energy coming from any direction to one of the rectifiers as shown in Fig. 1a. This subsection investigates the effect of varying the number of antenna ports *Na* and beam ports *Nb* in the Rotman lens on its maximum array factor and angular coverage. The (*Na*, *Nb*) set, resulting in the best combination, will define the Rotman lens design parameters used for this work. Structures of varying sizes were designed using Antenna Magus and identical material parameters (substrate, conductors) as the ones of the presented design, before being simulated in CST STUDIO SUITE 2018. The simulated data was then processed in MATLAB to output the array factors created by the respective lens structures using a modified version of Eq. (1)^12^, presented next in Eq. (2):

1 $$\begin{aligned} AF = \sum \limits _{n=1}^{Na} e^{j(n-1)(kdcos\theta + \beta )} \end{aligned}$$

where *AF*, *n*, *Na*, *k*, *d*, $\theta$ and $\beta$ are, respectively, the lossless array factor, the antenna number, the total number of antenna ports, the wave vector, the spacing between the elements, the direction of radiation and the difference in phase excitation between the elements. Since this formula describes a lossless array with a single antenna port, we introduced the following equation that takes into account the losses induced by the feeding network as well as the introduction of multiple feeding ports.

2 $$\begin{aligned} AF_j = \sum \limits _{n=1}^{Na} S_{nj} e^{j(n-1)(kdcos\theta )} \end{aligned}$$

where $AF_j$ and $S_{nj}$ are, respectively, the array factor for beam port *j* and the S parameters between antenna ports *n* and beam ports *j*. The maximum value of the array factors as well as their total (accounting for the aggregated coverage of all ports) 3 dB beamwidths where then tabulated. The five simulated lenses had the following (*Na*, *Nb*) combinations: (4,3), (8,6) representing the system implemented in this work, (16,12), (32,24) and (64,48). Figure 1b shows the increase in the array factor until reaching a peak of around 7.8 dB for a lens surrounded by 16 antennas and 12 beam ports, after which the array factor starts dropping, down to approximately 5.2 dB for a 64 antennas structure with 48 beam ports. The array factor reduction is explained by the increased losses within the lens accompanied by the increase of complexity and internal reflections, as the lens grows in electrical size. The same plot shows the decrease in angular coverage from 180° with 4 antennas down to 80° with 64 antennas. This study shows that the combination composed of eight antennas and six beam ports, offers a nearly optimal compromise, with these materials, between a high array factor of 5.95 dB and a 120° total angular coverage, while maintaining a reasonable number of antennas and beam ports. It should be noted that the choice of the number of beam ports is related to the 3dB-beamwidth of the individual antennas, the reason for which will be detailed later.

### Flexible broadband Rotman lens design

**Figure 2:**
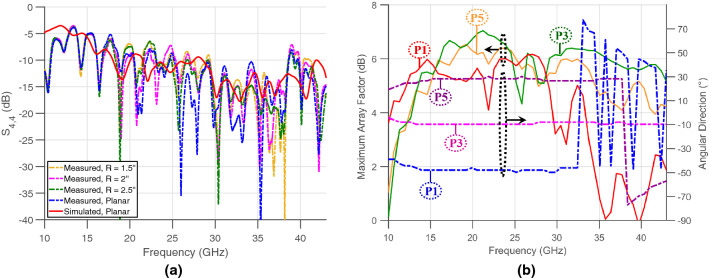
(**a**) Plot of the simulated and measured reflection coefficients at beam port 4 under planar and bent conditions and (**b**) Plots of the maximum array factors and angular directions of beam ports P1, P3 and P5 with respect to frequency.

After setting the number of antenna ports and beam ports, the design was printed on flexible copper-clad Liquid Crystal Polymer (LCP) substrate ($\varepsilon _r = 3.02$ and $\hbox {h}= 180\,\upmu \hbox {m}$) using an inkjet-printed masking technique followed by etching, resulting in the structure shown in Fig. 1c. It should be noted that the use of impedance-matched dummy ports is common with Rotman lenses^13–16^. Nevertheless, the goal in the implementation hereby described is not (as is usually the case) the generation of clean beam patterns with low side-lobe levels. Here, the lens’ properties are used for harvesting. Consequently, as long as the presence of the side lobes does not significantly interfere with the level of the array factor at broadside, side lobes are of no concern. Such a structure, including eight antenna ports and six beam ports—and, therefore, six radiating directions—was designed, simulated, and tuned. The structure, shown in Fig. 1c, with the antenna ports connected to matched loads, was then tested in planar and bent configurations—cylinders with different bending radii ranging from 1.5 to 2.5 in. radii—to assess the effect of bending on the S parameters behavior. Figure 2a shows the measured reflection coefficient of the Rotman lens at beam port 4 for four different scenarios, in comparison with the simulated structure in a planar position. The results reveal the Rotman lens’ ability to be mounted on curved surfaces down to a radius R = 1.5″, while maintaining a stable matching and minuscule losses compared to being held in a planar position.

The gain and angular bandwidths of this structure—defined by the frequency range in which the maximum array factor and angular direction per beam are stable within 3 dB and 5° respectively,—are studied next. The ultimate assessment of these properties involves calculating the beams’ magnitude and angular directions over a wide range of frequencies^17^, in order to ascertain their stability or lack thereof. For this purpose, the maximum array factors were calculated and the beams’ angular directions were extracted and plotted in Fig. 2b for the first, third and fifth beam ports, P1, P3 and P5, representing the edge, secondary and central beams in this symmetrical structure. These plots prove the unique capabilities offered by the Rotman lens; although the Rotman lens is designed at a specific frequency—28 GHz in this work—this analysis proves that both the magnitude and the angular direction of the beams remain relatively stable over a very wide frequency range. In Fig. 2b, three plots refer to the maximum array factors of the three beam ports, where minor fluctuations between 4 and 7 dB are observed over the range from 10 to 43 GHz for ports P3 and P5 and similar fluctuations over a fairly reduced frequency range for the extreme edge beam P1. On the same graph, three plots present the angular direction’s stability of P1, P3 and P5 beams, where P3 (in particular) preserves its angular direction over 33 GHz of bandwidth. The lens’ angular coverage resides between ports 1 and 6 and can be extracted from Fig. 2b. Knowing that the structure is symmetrical and that beam port P1 is at around ${-54}^\circ$, the overall structure covers an angle larger than 100° in front of the lens, a result further detailed in the next subsection. It should be noted that such a beamwidth is maintained over a large angular bandwidth exceeding 20 GHz, as shown in Fig. 2b. This study demonstrates the stability and robustness of a low-cost, printed and flexible mm-wave Rotman lens structure, tested with respect to bending and frequency, and supports the choice of such an architecture at the heart of the harvesting system proposed in this work.

### Flexible, high-gain and wide-angular-coverage mm-wave Rotman-lens-based antenna array

**Figure 3:**
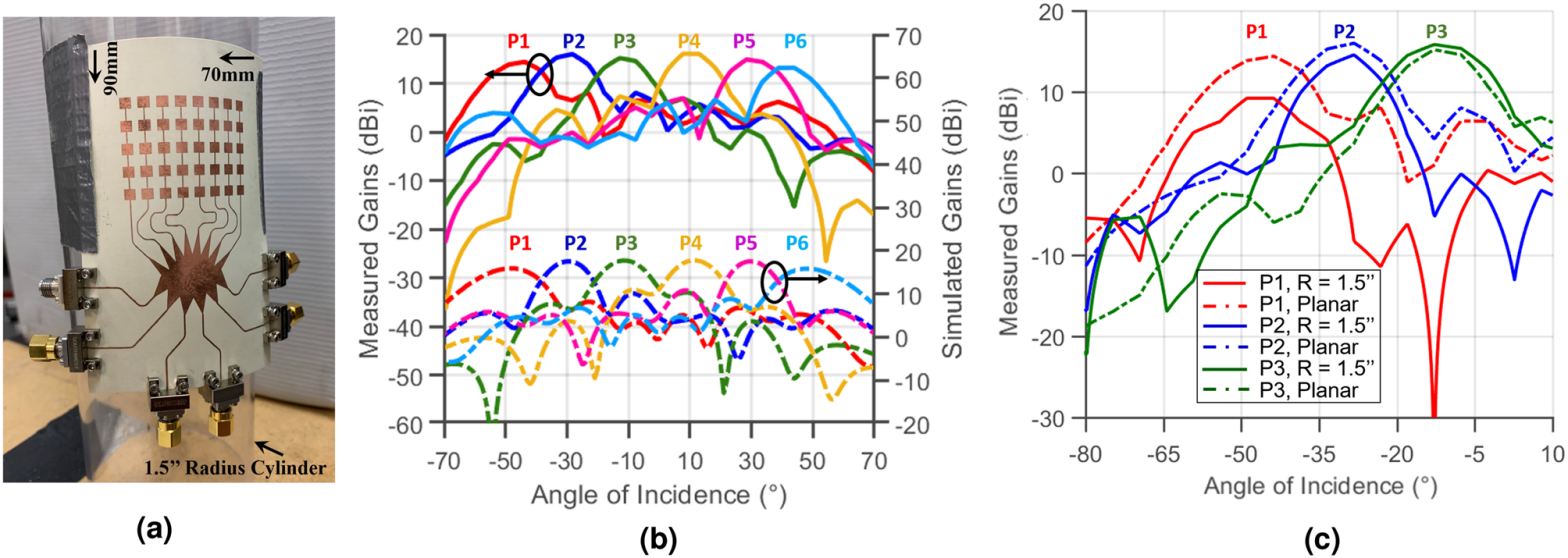
(**a**) Picture of the flexible Rotman-lens-based antenna array, (**b**) measured (solid lines) and simulated (dashed lines) gains of the antenna array held in a planar position and (**c**) measured gains of the antenna array for beams P1, P2 and P3 only (because of the symmetry of the structure) in planar and bent conditions.

Eight of the linear antenna sub-arrays introduced in^8^ were then added to the antenna ports of the array, and its beam-ports were extended by microstrip lines to enable their connection to end-launch ${2.92}\,\upmu \hbox {m}$ connectors. The antenna sub-array consists of five serially-fed patch antenna elements, providing an operation centered at 28.55 GHz with a reflection coefficient $S_{11}$ lower than ${-20}$ dB within this range. Their E-plane beamwidth of about ${18}^\circ$ (provided by the five antennas) is appropriate for most use cases, where environments expand mostly horizontally. Its simulations showed a gain of 13 dBi and a H-plane beamwidth of 80° in the plane perpendicular to the linear array. In this configuration, six beams were chosen to intersect at angles providing 3dB lower gain than broadside. Eight antennas provide a 3dB-beamwidth of 15°, which covers a total of $6\times {18}^\circ = {108}^\circ$ in front of the array. The design was then also printed on flexible LCP substrate, resulting in the structure shown in Fig. 3a, mounted on a 1.5″ radius cylinder. The radiation properties of the lens-based antenna system were simulated using the time-domain solver of CST STUDIO SUITE 2018, resulting in the six gain plots shown in Fig. 3b. The gain of the Rotman lens at every port was also accurately measured using a 20 dBi transmitter horn antenna and by terminating all five remaining ports with a ${50}\,\Omega$ load for every port measurement to guarantee the proper operation of the lens. Both simulated and measured radiation patterns (shown in Fig. 3b) display a remarkable similarity with a measured gain of approximately 17 dBi, and an angular coverage of around 110°, thereby validating the operation of the antenna array. The gains on the first three ports were also measured for the bent structure over a curvature of 1.5″ radius, shown in Fig. 3a and compared to the measured results on a planar surface. The previous subsection in addition to previous works^18,19^ have demonstrated that the performance of the Rotman lens is not deteriorated by wrapping or folding the structure compared to its conventional planar counterpart. However, after adding the antenna arrays, bending the structure can indeed have effects on its phase response, especially if the structure is large and the bending is severe. Figure 3c shows the gains of P1, P2 and P3 for the two scenarios (three ports only because the structure is symmetrical), demonstrating again the ability of the lens in maintaining a stable gain (especially over the center beams) upon bending. The beam located at the edge, however, suffers additional deterioration in received power under bending, because of the shift of the source away from the broadside of the bent antenna arrays.

### Fully-flexible 28 GHz Rotman lens-based system

#### Rotman-lens-based rectenna

**Figure 4:**
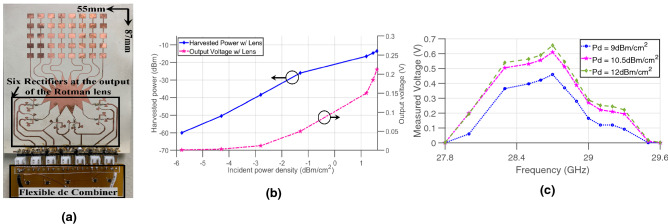
(**a**) Picture of the fully-flexible Rotman-based rectenna, (**b**) plot of the measured voltages and output powers versus incident power density for the Rotman-based rectenna and (**c**) plot of the measured voltages with respect to frequency for the Rotman-based rectenna.

In this section, the fully-flexible rectenna system—based on the Rotman lens and a new DC combiner network—is presented. This architecture, shown in Fig. 4a, consists of a series of eight antenna sub-arrays attached to the Rotman lens from one side, facing six rectifiers at the opposite side where DC serial combination is implemented. The basic rectenna elements, that are the antenna and the rectifier, are presented in details in^8^. The diode used in this work is the MA4E2038 Schottky barrier diode from Macom. The Rotman-based rectenna was first characterized as a function of its received power density. The system was positioned at a specific harvesting angle (approximately $-25^\circ$) and illuminated with a horn antenna with a gain of 20 dBi, placed at a distance of 52 cm away from the rectenna array, within the far field region starting at 23 cm, and outputting powers ranging from 18 to 25 dBm, corresponding to an RF input power sweep from around − 9 dBm to − 2 dBm. The array was loaded with its optimal load impedance of 1 $\hbox {k}\Omega$, corresponding to the optimal load of a single rectifier—since only one rectifier will be “ON” at a time, given that the Rotman lens focalizes all the power to one beam port depending on the direction of the incoming wave—as detailed earlier. The results of this experiment are shown in Fig. 4b, where the harvested voltages and powers of the array are shown. It can be observed that, at low powers, the Rotman-based rectenna effortlessly produces an output. The Rotman-based rectenna turns on well below − 6 dBm cm^−2^, which compares quite favorably to the literature^6^. The output voltage of the rectenna was also measured over its operating frequency range. Like in the first experiment, the system was positioned at the same harvesting angle, at a range of 25 cm away from the source’s horn antenna. The output voltages under open load conditions were recorded and plotted, as shown in Fig. 4c for the Rotman lens-based rectenna, for $P_d = {9}\,{\hbox{dBm cm}}^{-2}$, $P_d = {10.5}\,{\hbox{dBm cm}}^{-2}$ and $P_d = {12}\,{\hbox{dBm cm}}^{-2}$ incident power densities. The plots present a wide frequency coverage—from 27.8 to 29.6 GHz.

### Flexible DC combining network

Power summation is very critical when it comes to the unbalanced rectification outputs produced from realistic RF sources, and can be implemented differently depending on its costs and benefits^20^.

**Figure 5:**
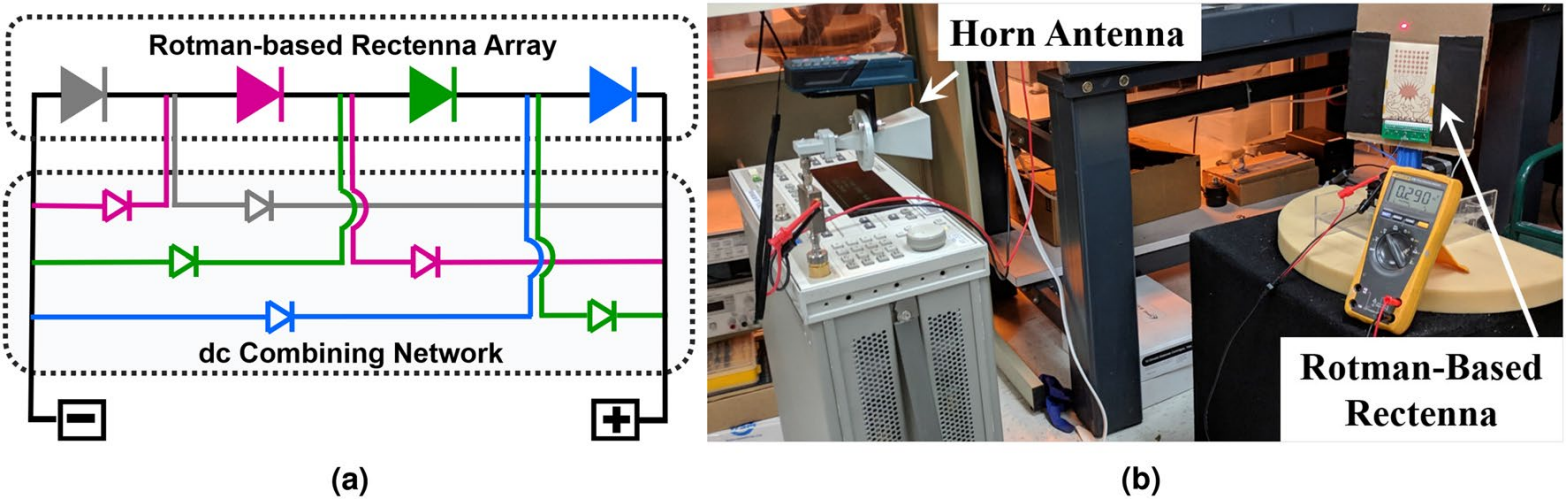
(**a**) Rotman-based rectenna power summation network and (**b**) picture of the setup used to measure the angular response of the rectenna.

**Figure 6:**
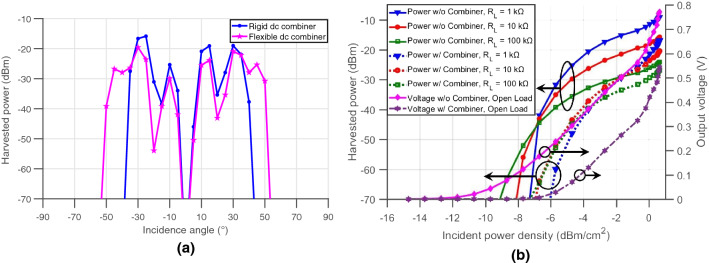
(**a**) Plot of the measured harvested powers by the rectenna with respect to the source’s incidence angle for the two DC combiners, rigid and flexible and (**b**) plots of the measured harvested powers and voltages with respect to the incident power density under different load conditions for the Rotman lens rectenna with and without the flexible DC combiner.

This paper does not rely on a direct voltage summation topology (i.e. back-to-back RF diodes); however, it introduces a minimalist architecture relying on a total of $2\times (N-1)$ bypass diodes, where *N* is the number of RF or rectifying diodes. Equipped with a low turn-on voltage of 0.1 V, the Toshiba 1SS384TE85LF bypass diodes used in the DC combiner design create a low resistance current path around all other rectifiers that received very low or close to zero RF power. This topology is optimal when only one diode is turned on, which can be assumed if a single, dominant source of power irradiates this particular design from a given direction. This new combiner circuit is shown in the schematic of Fig. 5a. This simplified schematic—shown for four rectifying diodes—uses different colors to highlight the paths that the current will take for every case where an RF diode turning “ON” while the serially-connected diodes are “OFF”. This DC combiner was then fabricated on a flexible ${125\,\upmu \mathrm{m}}$-thin polyimide Kapton substrate and connected to the Rotman lens-based rectenna through a series of single connectors to make the entire system fully flexible and bendable. The harvested power under a load of 1 $\hbox {k}\, \Omega$ versus the angle of incidence of the mm-wave energy source for the Rotman-lens-based rectenna is compared for both rigid (presented in^8^, and relying on $2\times N$ bypass diodes) and flexible new DC combiners. For this experiment, a horn transmitter antenna was used to send 25 dBm of RF power at 28.5 GHz to the lens placed 70 cm away, as shown in Fig. 5b, while the array was precisely rotated in angular increments of 5°. Figure 6a shows that the new DC combiner, with a reduced number of diodes, was able to provide a complete angular coverage of almost 110° over the entire lens spectrum as presented in Fig. 3b, thus solving the voltage nulling occurring at the first and last ports, using the rigid DC combiner adopted previously in^8^. The new DC combiner offers therefore, an increase of more than 30% in the system’s spatial angular in addition to enabling a fully-bendable structure due to the unique fabrication on flexible Kapton substrate and connection to the rectenna using individual interconnects.

As mentioned earlier, the DC combiner is mainly used with the Rotman-lens-based rectenna to automatically direct the active rectifier’s output to a single DC common port, independent of which port this might be. An alternative to the DC combiner in the Rotman lens-based system, would be to manually connect to the active port if the location of the source were known. To study the effect of the implemented DC combiner on the turn-on sensitivity of the system, the output voltage of the rectenna was measured for a specific source location with and without the combiner over a range of RF transmitted power and load variations; the direction was chosen such that the non-DC-combined rectifier would output its maximum power. Figure 6b shows eight different plots where three of them represent the harvested power with a direct connection to the active rectifier for 1 $\hbox {k}\Omega$, 10 $\hbox {k}\Omega$ and 100 $\hbox {k}\Omega$ conditions. Plotted with the same colors are the other three, representing the harvested power with the addition of the DC combiner for the same load values. The last two plots display the measured voltages with and without the combiner under open load conditions. The rectenna was placed 61 cm away from the transmitter horn antenna and the power was swept from 10 to 25 dBm. The results show the performance superiority in all considered load conditions when the contact is made directly to the rectifier and not through the DC combiner. The lens-based system is able to achieve a turn-on power as low as $-15\,{\hbox{dBm cm}}^{-2}$ in this case. This behavior is explained by the voltage drop introduced by the bypass diodes present in the combiner—that consistently decrease the expected output voltage by 0.1 to 0.2 V—when one or two diodes are, respectively, added to the current path. The variation of load values also shows that the rectenna can achieve better efficiencies at lower loads. More importantly, the reduction in the turn-on sensitivity—the minimum power density required output 10 mV—induced by the combiner is only of about 2 dB in loaded conditions, while the combiner enables an increase in the angular coverage of the rectenna system from about 18° to 110°. The remarkable angular and high-power turn-on sensitivity offered by the Rotman-lens-based rectenna are finally benchmarked using the following table for comparison with several state-of-the-art works, as presented in literature. In Table 1, the striking performance of the proposed system is displayed, highlighted by its flexibility and ability of achieving an angular coverage as large as 110° at extremely high turn-on sensitivity, thereby allowing mm-wave long-range harvesting in ad-hoc and conformal-mounting implementations.

**Table 1:** Performance comparison.

Ref.	Frequency (GHz)	Rectenna turn-ON power density	Rectenna angular coverage (°)	Flexible
^5^	24	5 dBm cm^−2^ at $R_L = {739}\,{\Omega }$	40	YES
^6^	24	− 3 dBm cm^−2^ at $R_L = {160}\,{\Omega }$	40	NO
^7^	20.2	− 3 dBm cm^−2^ at $R_L = {300}\,{\Omega }$	40	NO
^8^	28	− 6 dBm cm^−2^ at $R_L$ = 1 $\hbox {k}\Omega$	85	NO
This work	28	− 6 dBm cm^−2^ at $R_L$ = 1 $\hbox {k}\Omega$	110	YES

### Rectenna system performance under bending

**Figure 7:**
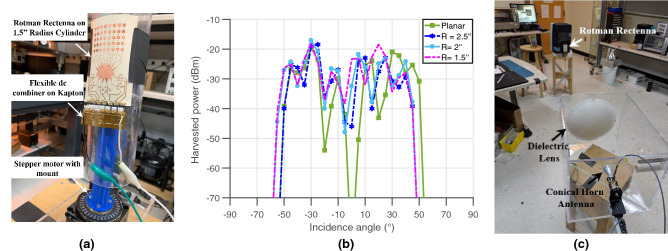
(**a**) Picture of the flexible Rotman lens-based rectenna placed on a 1.5″ radius cylinder and (**b**) measured harvested powers versus incidence angles for different curvatures, (**c**) long-range harvesting testing setup.

This section displays the operation of the Rotman-lens-based system under different bending scenarios. This and previous work^18,19^ show that the lens is able to maintain an efficient electromagnetic energy distribution across the output ports under convex and concave flexing conditions. The lens-based rectenna was placed on cylinders with different curvatures, 70 cm away from the transmitter sending 25 dBm of power at 28.5 GHz, as shown on Fig. 7a. The voltage was collected using a load of 1 $\hbox {k}\Omega$ for the planar and three bent conditions and plotted in Fig. 7b with respect to the source’s angle of incidence. The graph shows an unprecedented consistency and stability in the system’s scavenging and rectification abilities, knowing that several sub-systems are exposed to warping and the pressures of bending: the antenna sub-arrays, the Rotman lens and the rectifiers. Slight attenuation can be observed at the edges, but the system otherwise performs unimpeded by the bending. This remarkable property qualifies this system as a perfect candidate for use in wearables, smart phones and ubiquitous, conformal 5G energy harvesters for IoT nodes.

### Long-range harvesting

As described earlier, one of the main appeals of the proposed approach is its ability to use the high EIRPs allowed for 5G base-stations while guaranteeing an extended beam angular coverage, which is a necessary feature for ad-hoc ubiquitous harvesting implementations. In order to demonstrate the lens based-rectenna for longer-distance harvesting and detect that maximum range, a high-performance antenna system—comprised of a 19 dBi conical horn antenna and a 300 mm-diameter PTFE dielectric lens (for high directivity) providing an additional 10 dB of gain—was used as shown in Fig. 7c. With a transmitted power of 25 dBm (and an associated EIRP of approximately 54 dBm), corresponding to an incident power density of approximately − 6 dBm cm^−2^, the lens-based rectenna displayed an extended range of 2.83 m under open load conditions, with an output voltage around 10 mV, thereby demonstrating (to our knowledge) the longest-ranging rectenna demonstration at mm-wave frequencies. With a transmitter emitting the allowable 75 dBm EIRP, the theoretical maximum reading range of this rectenna could extend to 16 m. In addition, the use of advanced diodes—designed for applications within the 5G bands and enabling rectifiers’ sensitivities similar to that common at lower (UHF) frequencies—are showing a potential path towards achieving a turn-on sensitivity of the rectifiers as low as − 30 dBm^21,22^. If this were practically applied to the Rotman lens system presented in this work, the harvesting range could be extended beyond 180 m (where the received power density for a transmitted power of 75 dBm is ${7.8}\,\upmu \hbox {W cm}^{-2}$), which is only slightly smaller than the recommended cell size of 5G networks^23^. This observation enables the striking idea that future 5G networks could be used not only for tremendously-rapid communications, but also as a ubiquitous *wireless power grid* for IoT devices.

## Conclusion

Through the use of the Rotman lens, this paper demonstrates that the usual paradigm constrained by the (often considered fundamental) trade-off between the angular coverage and the turn-on sensitivity of a wireless harvesting system can be broken. Using the reported architecture, one can design and fabricate flexible mm-wave harvesters that can cover wide areas of space while being electrically large and benefit from the associated improvements in link budget (from source to harvester) and, more importantly, turn-on sensitivity. The approach has been shown, however, to only be scalable up to the degree where the additional incremental losses introduced by the growing lens counterbalance the increase in the aperture of the rectenna. Nevertheless, this inflection point only appears (in the particular context considered in this paper) after the arraying of 16 elements, or up to a scale of $8\lambda$. In the 5G Frequency Range 2 (FR2), this translates to harvesters of 4.5 cm to 9.6 cm in size, which are perfectly suited for wearable and ubiquitous IoT implementations. With the advent of 5G networks and their associated high allowed EIRPs and the availability of diodes with high turn-on sensitivities at 5G frequencies, several ${\upmu \hbox {W}}$ of DC power (around 6 ${\upmu \hbox {W}}$ with 75 dBm EIRP) can be harvested at 180 m. Such properties may trigger the emergence of 5G-powered nodes for the IoT and, combined with the long-range capabilities of mm-wave ultra-low-power backscatterers^24^, of long-range passive mm-wave RFIDs.
